# Fabrication of an NIR-II fluorescence-visible indocyanine green-BioGlue for precision surgical adhesion with intraoperative leakage prevention

**DOI:** 10.1093/rb/rbaf131

**Published:** 2025-12-24

**Authors:** Xinyu Feng, Ruihan Liu, Cai Zhang, Ran Cheng, Dianxun Fu, Shaokai Sun, Junping Wang, Guohe Wang

**Affiliations:** School of Medical Imaging, Division of Medical Technology, Tianjin Key Laboratory of Functional Imaging, Tianjin Medical University, Tianjin 300203, China; Department of Breast Imaging, Tianjin Medical University Cancer Institute & Hospital, National Clinical Research Center for Cancer, Tianjin’s Clinical Research Center for Cancer, Key Laboratory of Breast Cancer Prevention and Therapy, Tianjin Medical University, Ministry of Education, Key Laboratory of Cancer Prevention and Therapy, Tianjin 300060, China; School of Medical Imaging, Division of Medical Technology, Tianjin Key Laboratory of Functional Imaging, Tianjin Medical University, Tianjin 300203, China; Department of Radiology, Tianjin Medical University Cancer Institute and Hospital, National Clinical Research Center for Cancer, Tianjin’s Clinical Research Center for Cancer, Key Laboratory of Cancer Immunology and Biotherapy, Tianjin 300060, China; School of Medical Imaging, Division of Medical Technology, Tianjin Key Laboratory of Functional Imaging, Tianjin Medical University, Tianjin 300203, China; Department of Radiology, Tianjin Key Laboratory of Functional Imaging, Tianjin Medical University General Hospital, Tianjin 300052, China; School of Medical Imaging, Division of Medical Technology, Tianjin Key Laboratory of Functional Imaging, Tianjin Medical University, Tianjin 300203, China; Department of Radiology, Tianjin Key Laboratory of Functional Imaging, Tianjin Medical University General Hospital, Tianjin 300052, China; School of Medical Imaging, Division of Medical Technology, Tianjin Key Laboratory of Functional Imaging, Tianjin Medical University, Tianjin 300203, China

**Keywords:** BioGlue, Bioadhesives, NIR-II fluorescence imaging, intraoperative leakage prevention, surgery navigation

## Abstract

Bioadhesives are being increasingly used in clinical practice, but pose significant health risks due to their uncontrollable intraoperative leakage. Nevertheless, there is currently a critical lack of effective solutions. Herein, we propose a second near-infrared window (NIR-II) fluorescence-visible indocyanine green (ICG)-BioGlue adhesive for precision surgical adhesion with intraoperative leakage prevention. The fabricated ICG-BioGlue adhesive based on the non-covalent interaction exhibits nearly 100% fluorescence labeling efficiency and maintains long-term stable NIR-II fluorescence for up to 3 months. During surgery, ICG-BioGlue adhesive enables real-time visualization of wound adhesion in various fields such as aortic dissection adhesion and liver or kidney wound hemostasis, preventing leakage into surrounding tissues. Moreover, the stable fluorescence of ICG-BioGlue adhesive allows effective imaging-guided removal of long-term adhesive fragments in the body when they cause compression symptoms after surgery. Considering it consists solely of Food and Drug Administration-approved drugs ICG and BioGlue, with a straightforward synthesis method and versatile applicability, ICG-BioGlue adhesive holds excellent potential for clinical translation. Our study provides a leakage-preventing strategy to enhance biosafety and promote the widespread application of bioadhesives in clinical settings.

## Graphical abstract

**Figure:**
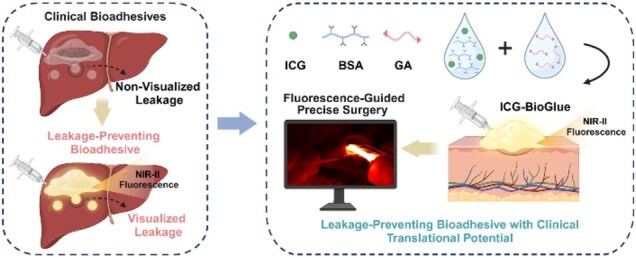

## Introduction

Bioadhesives have emerged as a preferred alternative to traditional surgical sutures and staples in wound management, owing to the advantages of convenience, reduced trauma, faster healing, lower infection risk, less pain, versatility and reduced postoperative care [1, 2]. So far, various bioadhesives, represented by BioGlue (Cryolife, USA), composed of glutaraldehyde (GA) and purified bovine serum albumin (BSA) solution, have been approved by the Food and Drug Administration (FDA) and widely used in clinical practice [3–5]. BioGlue adhesive is the most commonly used biological adhesive in cardiovascular surgery and is also employed for controlling air leaks following lung surgeries, sealing the dura mater in neurosurgical procedures, and achieving hemostasis during nephron-sparing surgeries [6–9]. However, bioadhesives often risk intraoperative leakage into surrounding tissues, potentially causing life-threatening embolism [10–14]. Moreover, bioadhesives can induce “glue-oma” after surgery, causing local inflammation and compression that may require additional surgery to remove and alleviate patient discomfort [15–17].

Preventing the occurrence of various complications is significantly more crucial than their subsequent diagnosis and treatment. Unfortunately, various imaging techniques (such as computed tomography, magnetic resonance imaging and ultrasound imaging) are limited in assessing postoperative complications of bioadhesives and fail to provide real-time visualization of adhesive application during surgery to mitigate the risk of complications [16, 18–20]. Fluorescence imaging, with advantages of high sensitivity, non-ionizing radiation and high temporal resolution, has become a powerful tool in current clinical surgical navigation, especially with the second near-infrared window (NIR-II) imaging offering superior spatial and temporal resolution along with deeper tissue penetration [21–29]. However, previous studies have mainly focused on two directions. One direction involves the use of fluorescent probes to detect lesions and their associated microenvironment [30–39], and the other involves labeling adhesives with fluorescent probes to trace the adhesives and evaluate their degradation behavior in tissue engineering and drug delivery applications [40–45]. Despite these advances, a critical knowledge gap remains regarding how to achieve real-time intraoperative visualization of BioGlue in order to prevent the potential risk of unintended adhesive leakage.

**Figure 1:**
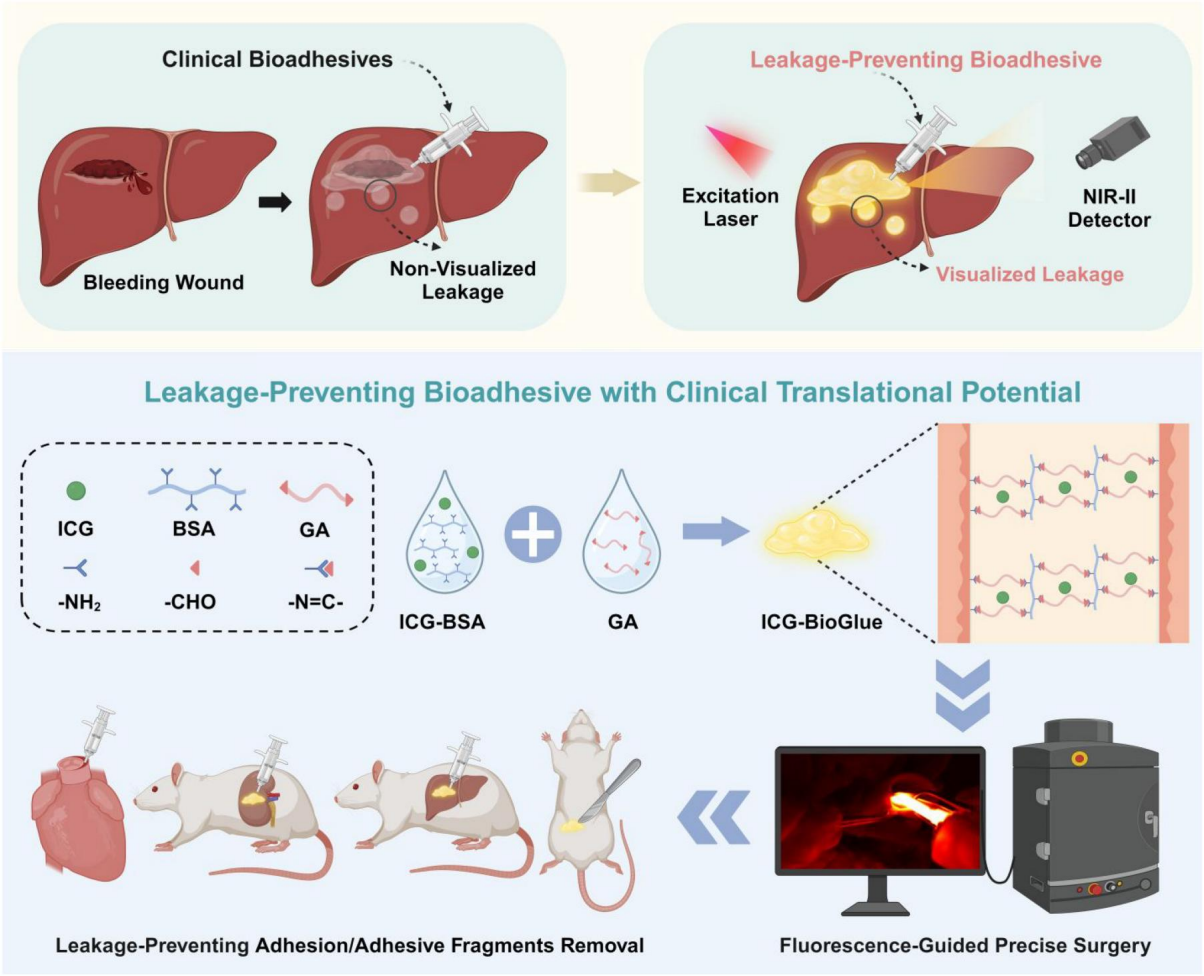
Schematic illustration of the ICG-BioGlue adhesive for the NIR-II fluorescence-guided leakage-preventing surgical adhesion *in vivo*.

Herein, we propose an NIR-II fluorescence-visible indocyanine green (ICG)-BioGlue adhesive for precision surgical adhesion with intraoperative leakage prevention. The ICG-BioGlue adhesive was fabricated based on the non-covalent interaction by stirring BSA (45 wt%) and ICG at room temperature for 1 h, followed by uniform mixing with GA (10 wt%) to rapidly generate an adhesive exhibiting intense NIR-II fluorescence. The proposed ICG-BioGlue adhesive showed nearly 100% fluorescence labeling efficiency and exhibited long-term stable NIR-II fluorescence for up to 3 months. The ICG-BioGlue adhesive achieved effective NIR-II fluorescence imaging guidance in sealing aortic dissection and liver and kidney wounds, thereby reducing the risk of adhesive leakage. In addition, ICG-BioGlue adhesive with long-term stable NIR-II fluorescence (at least 3 weeks *in vivo*) enabled efficient adhesive fragment removal surgery. More importantly, the proposed ICG-BioGlue adhesive, featuring a straightforward synthesis process and FDA-approved components, exhibited significant clinical translational potential. This work offered an elegant approach to preventing intraoperative adhesive leakage and facilitated the clearance of postoperative adhesive fragments, thereby promoting the safer and wider application of bioadhesives (Figure 1).

## Materials and methods

### Materials

All chemicals used in our experiments adhered to analytical-grade purity standards. BSA, GA, and ICG were procured from Aladdin Reagent Co., Ltd (Shanghai, China). Dimethyl sulfoxide (DMSO) was gained from Concord Technology (Tianjin, China). Ultrapure water was purchased from Wahaha Group Co., Ltd (Hangzhou, China).

### Characterization

Fourier transform infrared (FT-IR) spectra were obtained using a Nicolet iS10 spectrometer (Nicolet, USA). Scanning electron microscopy (SEM) was utilized to capture microstructure images (Zeiss Gemini 300, Germany). UV-3600 plus UV-Vis-NIR spectrophotometer (Shimadzu, Japan) was utilized for recording absorption spectra, while NIR-II fluorescence spectra were obtained using a fluorescence spectrometer (NIRQuest512, Ocean Optics, USA). The MTT assay and hemolysis test were carried out by a microplate reader (Bio-Tek, USA).

### Synthesis of ICG-BioGlue and BioGlue adhesive

Initially, BSA was exposed to ultraviolet irradiation for 1 h, followed by dissolution in ultrapure water with a concentration of 450 mg/mL. ICG was then added to the BSA solution and stirred for 1 h at room temperature. Subsequently, a 10 wt% GA solution was introduced to the mixture to fabricate the ICG-BioGlue adhesive, achieving a final ICG concentration of 100 μg/mL. The formation of the BioGlue adhesive involved blending the BSA solution with the GA solution at a volume ratio of 4:1.

### *In vitro* fluorescence imaging

The acquisition of NIR-II fluorescence images was performed using a custom-built small animal NIR-II imaging system, including an InGaAs camera (Princeton Instruments, NIRvana: 640, USA) with a fiber-coupled 808-nm laser (Changchun New Industries Optoelectronics Technology Co., Ltd) and a 980-nm long-pass filter. The fluorescence penetration depth of ICG-BioGlue adhesive was measured through NIR-II fluorescence imaging (100 ms, 53 mW/cm^2^). Adhesive blocks with lengths, widths, and thicknesses of 15, 10 and 5 mm were prepared and irradiated under chicken and beef tissues with different thicknesses (0, 1, 2, 3, 4 and 5 mm). Besides, the NIR-II fluorescence stability and labeling efficiency of ICG-BioGlue adhesive were assessed by immersing adhesive blocks (with a side length of 5 mm) in water or phosphate buffered saline (PBS) and maintaining them at either 4°C or 25°C, respectively. The fluorescence intensity of the supernatant was measured at various time points within 48 h to analyze the release rate of ICG (100 ms, 53 mW/cm^2^). NIR-II fluorescence images and photos of each block at varying time points (up to 100 days) were collected, and the changes in fluorescence intensity of the adhesive blocks were analyzed (100 ms, 11 mW/cm^2^).

### Tensile and rheological tests

The aortic vascular wall tissues from pigs were cut into pieces, each measuring 20 mm in length and 10 mm in width. Subsequently, 100 µL of either ICG-BioGlue or BioGlue adhesive was injected between the two tissue segments to bond them together. After a 10-minute standing period, the bonded tissues were pulled from both ends, and the maximum tensile strength was measured using a tensile tester (Suce, China). Rheology experiments of ICG-BioGlue and BioGlue adhesive were conducted on an Anton Paar xx2 modular compact rheometer (MCR). The dynamic oscillatory time sweep was measured with a strain amplitude set at 1% and an angular frequency fixed at 1 rad/s.

### Evaluation of adhesion performance and durability

The ICG-BioGlue adhesive was applied *in situ* onto fresh porcine skin and subjected to various deformation tests, including compression, stretching, and twisting. In addition, freshly excised rat organs were adhered to glass slides, and the slides were inverted after 30 s to assess the tissue adhesiveness of the ICG-BioGlue adhesive. Furthermore, a universal testing machine (WDW-2, Songdun, China) was used to perform uniaxial tensile tests to evaluate the adhesive performance of ICG-BioGlue and BioGlue. Porcine skin (80 × 25 mm^2^) was used as the tissue substrate, with an overlapping adhesion area of approximately 50 × 25 mm^2^ and a tensile rate of 5 mm/min. Subsequently, 100 cycles of loading tests were conducted on skin samples bonded with ICG-BioGlue and BioGlue under a stress of 1 N/cm^2^ and a tensile rate of 5 mm/min.

### Cellular cytotoxicity

ICG-BioGlue adhesive cytotoxicity was evaluated using the 3-(4,5-dimethylthiazol-2-yl)-2,5-diphenyltetrazolium bromide (MTT) method, with cells cultured in DMEM (Dulbecco’s Modified Eagle’s Medium) supplemented with 5% fetal bovine serum and 1% penicillin-streptomycin. Firstly, the freeze-dried ICG-BioGlue adhesive was soaked in the culture medium with different concentrations (0, 1, 2, 3, 4, 5, 6, 8, 10 12 mg/mL) and incubated for 24 h at 37°C to obtain the ICG-BioGlue adhesive extracts. In addition, mouse embryonic fibroblasts (3T3-L1 cells), rat cardiomyocytes (H9C2 cells) and human umbilical vein endothelial cells (HUVECs) were plated in 96-well plates at a density of 5 × 10^4^ cells per well and maintained at 37°C in a 5% CO_2_ environment. Following a 12-h incubation period, the culture medium was substituted with extracts of ICG-BioGlue adhesive with different concentrations, and cells were further incubated for 24 h. Following the addition of MTT solution (5 mg/mL, 10 μl) to each well, a 4-h incubation period was allotted, and purple formazan crystals were dissolved in DMSO. Afterwards, the absorbance of each well at 490 nm was quantified to evaluate cytotoxicity.

### Hemocompatibility analysis

The hemocompatibility of the ICG-BioGlue adhesive was assessed through hemolysis tests. Initially, freeze-dried ICG-BioGlue adhesive was soaked in normal saline (NS) with different concentrations (5, 10, 20, 40, 80, 120, 160 200 mg/mL) and maintained at a temperature of 37°C for a duration of 24 h to obtain ICG-BioGlue adhesive extracts. Afterwards, red blood cells were collected from Sprague-Dawley (SD) rat blood, washed with NS (2000 rpm, 5 min), and then suspended in water, NS, and ICG-BioGlue extracts, and incubated for 2 h at 37°C. The supernatant was obtained following centrifugation (10000 rpm, 5 min), and the absorbance at 540 nm was recorded subsequently.

### Animal models

All animal experiments were approved by the Animal Ethics Committee of Tianjin Medical University Cancer Institute and Hospital and were conducted according to the Guidelines for Care and Use of Laboratory Animals of Tianjin Medical University Cancer Institute and Hospital (AE-2022062). All Kunming mice weighing 20–25 g and SD rats weighing 180–200 g were procured from SPF Biotechnology Co., Ltd (Beijing, China). In addition, all animal procedures were performed under anesthesia induced by a small animal gas anesthesia machine, utilizing a blend of 0.5 L/min O_2_ gas and 3% isoflurane. The pig hearts (bought from the markets) were used to establish the *ex vivo* model of aortic dissection. The intima and media of the aortic root were separated with forceps to form a cavity, simulating the states during the type A aortic dissection root surgery. The *in vivo* liver hemostasis model was built in anesthetized rats positioned supine. Utilizing a scalpel, an incision was made through the skin and muscle to unveil the left lobe of the liver. Subsequently, a 10-mm-long and 3-mm-deep incision was carefully formed on the left lobe of the liver. The rat kidney hemostasis model was established using a similar approach. Anesthetized and depilated rats were positioned prone. The left kidney was exposed, and a circular wound with a diameter of 2 mm was created using a scalpel. Real-time monitoring of the adhesive *in vivo* was achieved by subcutaneously injecting mice. Following anesthesia, the abdomen of the mice was depilated, and then a cut was made in the skin at the right inguinal area. Subsequently, 100 μL of ICG-BioGlue adhesive was injected into the incision, simultaneously bonding the wound. The ICG-BioGlue adhesive removal was performed under the guidance of NIR-II fluorescence imaging, and the wound was sutured after surgery.

### *In vivo* biosafety analysis

All rats and mice were allocated into experimental groups and control groups randomly (*n* = 3). The experimental group rats were administered a 100-μL injection of ICG-BioGlue adhesive onto the surface of the left lobe of the liver, while rats in the control group were administered an equal volume of NS. In the experimental group of mice, a 100-μL injection of ICG-BioGlue adhesive was administered into the right inguinal incision, followed by wound closure. The control group mice were injected with an equivalent volume of NS, followed by suturing of the wounds. The weights of both experimental and control groups were monitored every 2 days. Animals were sacrificed post-operation after 1, 2 and 3 weeks, and their organs (heart, liver, spleen, lungs and kidney) were harvested for histopathological analysis. Blood samples from the animals were processed for biochemical analysis concurrently.

### Statistical analysis

The data were presented as means ± standard deviation and were statistically analyzed using GraphPad Prism 8.0. Statistical significance was determined using one-way analysis of variance, independent sample *t*-tests and paired *t*-tests. Significance was defined as *P < 0.05, **P < 0.01, ***P < 0.001, ****P < 0.0001.

## Results

### Synthesis and characterization of ICG-BioGlue adhesive

ICG is an FDA-approved near-infrared fluorescent dye that has been widely used in clinical practice for liver function assessment and fluorescence-guided surgery [46–48]. The preparation of the ICG-BioGlue adhesive involved mixing a BSA solution (450 mg/mL) with ICG and stirring the mixture at room temperature for 1 h. Following this, GA solution (10 wt%) was added to the mixture at a volume ratio of 4:1. The optimal content of ICG in the system was determined first. The absorption spectrum of an ICG solution was analyzed, revealing a distinct absorption peak at around 779 nm (Supplementary Figure S1A). The NIR-II fluorescence spectrum of the ICG solution was then measured, excited by an 808-nm laser, and the fluorescence emission wavelength extended to about 1200 nm (Supplementary Figure S1B). As the ICG content in the BSA solution increased, the color of the mixture transitioned gradually from light brown to deep green, while retaining its fluidity. When GA was added to the above mixture, the resulting solutions with varying ICG contents quickly solidified, indicating the formation of the ICG-BioGlue adhesive (Supplementary Figure S2A and B). With the addition of 100 μg/mL of ICG, the NIR-II fluorescence intensity of the ICG-BioGlue adhesive reached its highest point. However, as the amount of ICG exceeded 100 μg/mL, the fluorescence intensity of the adhesive decreased. These results were consistent with the analysis of the NIR-II fluorescence signals (Supplementary Figure S2C and D). Thus, the composition of the ICG-BioGlue adhesive was determined, introducing 100 μg/mL of ICG (final concentration in the ICG-BioGlue adhesive) to ensure the optimal fluorescence imaging effects.

**Figure 2:**
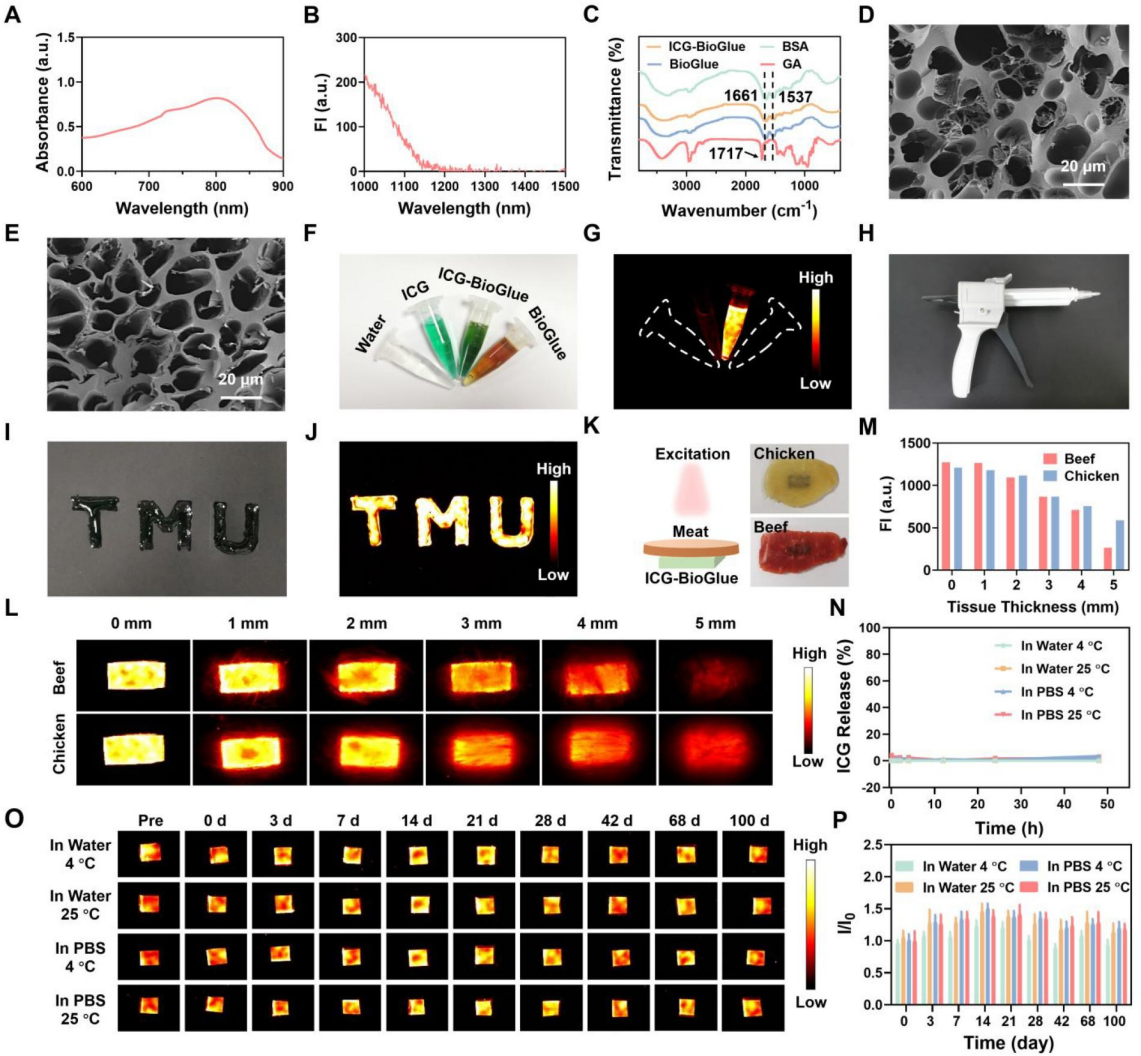
Characterization of ICG-BioGlue adhesive. (**A**) Absorbance spectrum and (**B**) NIR-II fluorescence spectrum of ICG-BioGlue adhesive. (**C**) FT-IR spectra of ICG-BioGlue adhesive, BioGlue adhesive, BSA, and GA. SEM images of (**D**) BioGlue adhesive and (**E**) ICG-BioGlue adhesive. Scale bars, 20 µm. (**F**) Photograph and (**G**) NIR-II fluorescence image of water, ICG, ICG-BioGlue adhesive, and BioGlue adhesive. (**H**) Custom cartridge delivery system of ICG-BioGlue adhesive. (**I**) Photograph and (**J**) NIR-II fluorescence image of a specific pattern (TMU) written with ICG-BioGlue adhesive. (**K**) Schematic diagram of the evaluation of the fluorescence penetration depth of ICG-BioGlue adhesive. (**L**) NIR-II fluorescence penetration depth of ICG-BioGlue adhesive covered with different thicknesses of chicken or beef tissues (0, 1, 2, 3, 4 and 5 mm). (**M**) NIR-II fluorescence intensity of images in (**L**). (**N**) ICG release rate of ICG-BioGlue adhesive in water or PBS at 4°C or 25°C within 48 h (*n* = 3). (**O**) Fluorescence stability of ICG-BioGlue adhesive in water or PBS at 4°C or 25°C for 100 days. (**P**) NIR-II fluorescence intensity of images in (O) (*n* = 5). FI, fluorescence intensity.

The absorption spectrum of ICG-BioGlue adhesive was then measured, and the absorption peak was redshifted to 801 nm (Figure 2A). The NIR-II fluorescence spectrum of the ICG-BioGlue adhesive was examined, which had strong fluorescence intensity in the NIR-II region (Figure 2B). FT-IR spectra showed the amide I band vibrations at 1661 cm^−1^ and amide II band vibrations at 1537 cm^−1^, which confirmed the presence of BSA in ICG-BioGlue adhesive [49]. Additionally, the characteristic aldehyde bands of GA at 1717 cm^−1^ disappeared, indicating the binding of aldehyde and amine groups during the reaction process (Figure 2C) [50]. The morphology of the ICG-BioGlue adhesive was characterized through SEM, and similar porous structures were observed in the ICG-BioGlue and BioGlue adhesive (Figure 2D and E). Then, tensile tests of the ICG-BioGlue and BioGlue adhesive were performed, which were injected between two pieces of aortic vascular wall tissues respectively. There was no significant disparity in the maximum tension endurance between the ICG-BioGlue and BioGlue adhesive, indicating that the introduction of ICG had no negative impact on BioGlue (Supplementary Figure S3). Besides, dynamic oscillatory time sweep measurements indicated that the ratio of storage modulus to loss modulus (G′/G″) of BioGlue and ICG-BioGlue adhesive was consistently >1 (Supplementary Figure S4). The results indicated that BioGlue and ICG-BioGlue adhesive possessed relatively high hardness. The ICG-BioGlue adhesive was formed *in situ* on fresh porcine skin and was able to withstand various deformations, including compression, stretching, and twisting, without peeling or cracking (Supplementary Figure S5A). In addition, freshly dissected rat organs were adhered to glass slides, and after 30 s, the slides were inverted to assess the tissue adhesiveness of ICG-BioGlue adhesive. The organs remained firmly attached to the glass slides even after inversion (Supplementary Figure S5B). Furthermore, a universal testing machine was employed to measure the adhesive strength of both ICG-BioGlue and BioGlue. No significant difference in adhesion strength was observed between the two adhesives (Supplementary Figure S6). Subsequently, cyclic loading tests (100 cycles) were performed on skin samples bonded with ICG-BioGlue and BioGlue adhesive. No noticeable fracture occurred during the tests, indicating that both ICG-BioGlue and BioGlue adhesive possess excellent fatigue resistance under dynamic conditions (Supplementary Figure S7).

*In vitro* fluorescence imaging performance of the ICG-BioGlue adhesive was evaluated by recording NIR-II fluorescence images. Significantly improved fluorescence intensity was observed with the ICG-BioGlue adhesive compared to pure ICG, due to the effective interaction between ICG and BSA (Figure 2F and G) [51–53]. The interactions between ICG and serum albumin have been extensively and deeply investigated. After intravenous administration, ICG rapidly binds to human serum albumin and is then cleared through the hepatobiliary pathway. Therefore, the concentration of ICG in blood can be used to reflect hepatic function [54]. ICG associates with BSA through multiple non-covalent interactions that include hydrophobic forces and electrostatic attraction. Once bound to albumin, ICG undergoes a transition from an aggregated state to a monomeric state, accompanied by an increase in molecular rigidity. This transition suppresses aggregation-induced quenching and nonradiative decay, which in turn enhances its fluorescence quantum yield and stability [55, 56]. The ICG-BioGlue adhesive was formed through a custom cartridge delivery system, ensuring uniform mixing of ICG-BSA and GA solutions at a 4:1 volume ratio (Figure 2H). The ICG-BioGlue adhesive exhibited controllable syringeability and could be injected into specific shapes as depicted in corresponding photographs and NIR-II fluorescence imaging (Figure 2I and J). To explore the fluorescence penetration depth of ICG-BioGlue adhesive, the adhesive blocks were placed under chicken or beef tissues with different thicknesses and irradiated with an 808-nm laser (Figure 2K). The fluorescence penetration depth of the ICG-BioGlue adhesive could reach 5 mm, and the NIR-II fluorescence intensity of the ICG-BioGlue adhesive under beef or chicken tissues gradually decreased as the thickness increased (Figure 2L and M). Furthermore, the ICG release rate and the fluorescence stability of ICG-BioGlue adhesive were examined by immersing the prepared adhesive blocks in water or PBS and keeping them at 4°C or 25°C, respectively. The ICG release rate was close to 0% within 48 h, indicating that the fluorescence labeling efficiency was close to 100% (Figure 2N). NIR-II fluorescence images of the adhesive blocks were captured at various time points over 3 months. Throughout the entire observation period, negligible changes in NIR-II fluorescence intensity and in the physical status of the adhesive blocks were observed, which can be attributed to the intrinsic structural stability of BioGlue adhesive and the strong interactions between ICG and BSA. These findings indicate the excellent fluorescence stability of the ICG-BioGlue adhesive (Figure 2O and P and Supplementary Figure S8). These results proved that ICG-BioGlue adhesive possessed nearly 100% fluorescence labeling efficiency and long-term stable NIR-II fluorescence for up to 3 months. These advantages made it possible for the ICG-BioGlue adhesive to be applied in the visualized wound adhesion or adhesive fragments during surgery under the NIR-II fluorescence imaging.

### Biosafety analysis of ICG-BioGlue adhesive

**Figure 3:**
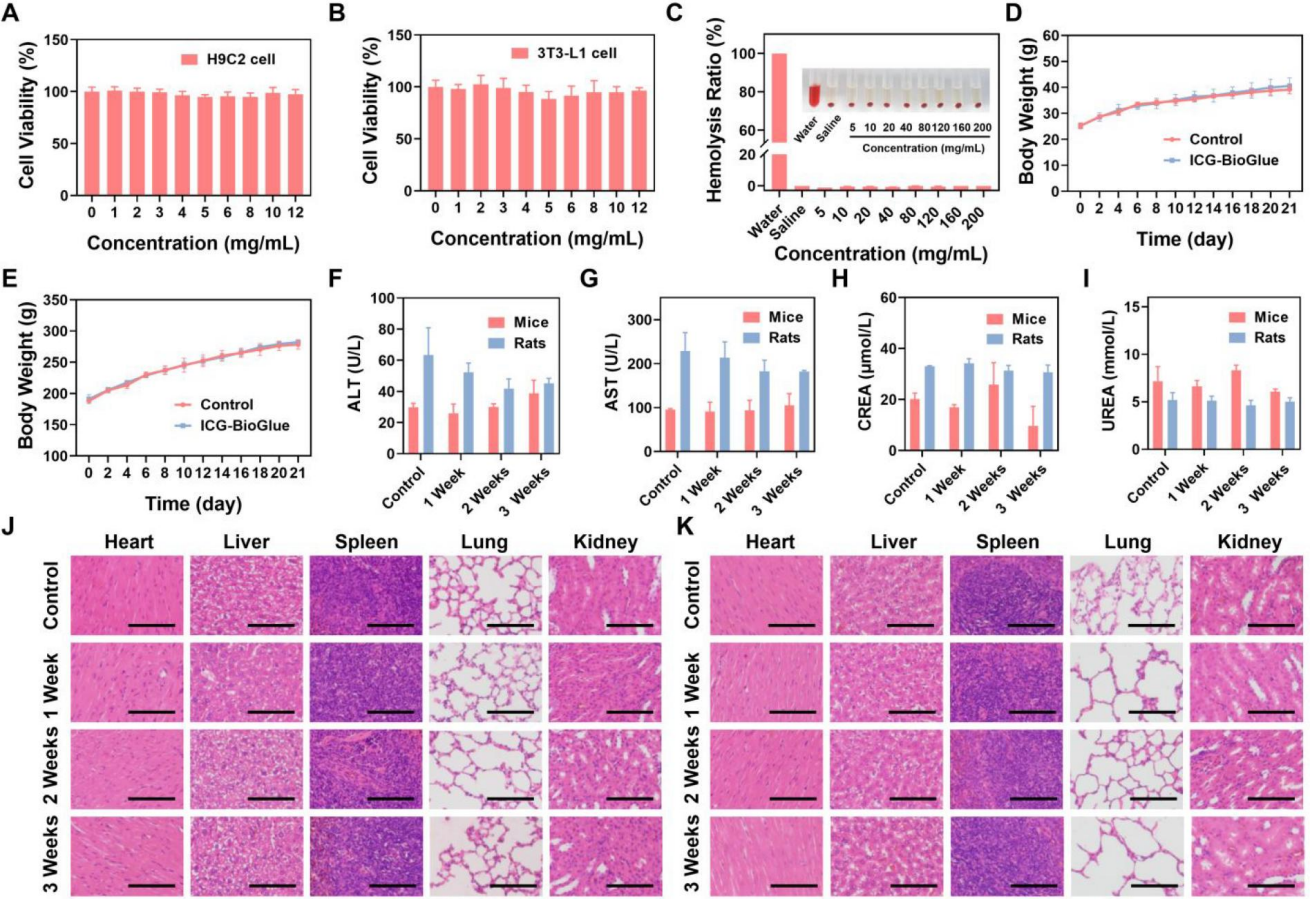
Biosafety evaluation of ICG-BioGlue adhesive. Cell viabilities of (**A**) H9C2 cells and (**B**) 3T3-L1 cells treated with different concentrations of ICG-BioGlue adhesive extracts (*n* = 6). (**C**) Photograph of the hemolysis test and hemolytic rates of ICG-BioGlue adhesive (*n* = 3). Body weight changes of (**D**) mice and (**E**) rats in the experiment group and control group for 3 weeks (*n* = 3). Blood biochemical indicators including (**F**) ALT, (**G**) AST, (**H**) CREA, and (**I**) UREA in the experiment group and control group for 3 weeks (*n* = 3). Histopathological images of major organs (hearts, lungs, livers, spleens, and kidneys) of (**J**) mice and (**K**) rats in the experiment group and control group for 3 weeks. Scale bars, 100 µm.

The cytotoxicity of the ICG-BioGlue adhesive was explored using the standard MTT method. H9C2 cells, 3T3-L1 cells, HUVECs were cultured with the media containing different concentrations of ICG-BioGlue adhesive extracts for 24 h. The cell viability was over 80% even though the concentration of ICG-BioGlue adhesive extracts reached 12 mg/mL, indicating negligible cytotoxicity of ICG-BioGlue adhesive (Figure 3A and B and Supplementary Figure S9). The hemocompatibility analysis of the ICG-BioGlue adhesive was further investigated through hemolysis experiments. Red blood cells were subjected to incubation with water, NS, and NS containing various concentrations of ICG-BioGlue adhesive extracts. Red blood cells incubated in water showed obvious hemolysis. Notably, no instances of hemolysis were observed in the groups of ICG-BioGlue adhesive with various concentrations. The hemolysis rate consistently remained below 5% even when the concentration of ICG-BioGlue adhesive extracts reached 200 mg/mL (Figure 3C). The above results confirmed the good *in vitro* biocompatibility of ICG-BioGlue adhesive.

The *in vivo* toxicity of the ICG-BioGlue adhesive was further investigated by randomly dividing all animals into experimental and control groups. A 100-μL injection of ICG-BioGlue adhesive was administered to the surface of the left lobes of the liver in rats or the right inguinal incision in mice. The rats and mice in the control groups received an equivalent volume of NS injection. No significant decrease in body weight was observed in either the experimental or control groups, and the common blood biochemical parameters such as alanine aminotransferase (ALT), aspartate aminotransferase (AST), creatinine (CREA), and urea (UREA) remained in normal ranges within 3 weeks after operations (Figure 3D–I). The main organs (hearts, livers, spleens, lungs and kidneys) collected at different time points (0, 1, 2 and 3 weeks) showed no significant histopathological damage (Figure 3J and K). Therefore, the ICG-BioGlue adhesive demonstrated good biosafety, and the injected volume of 100 μL was the safe dose for subsequent *in vivo* applications.

### *Ex vivo* aortic dissection repair surgery guided by fluorescence imaging

**Figure 4:**
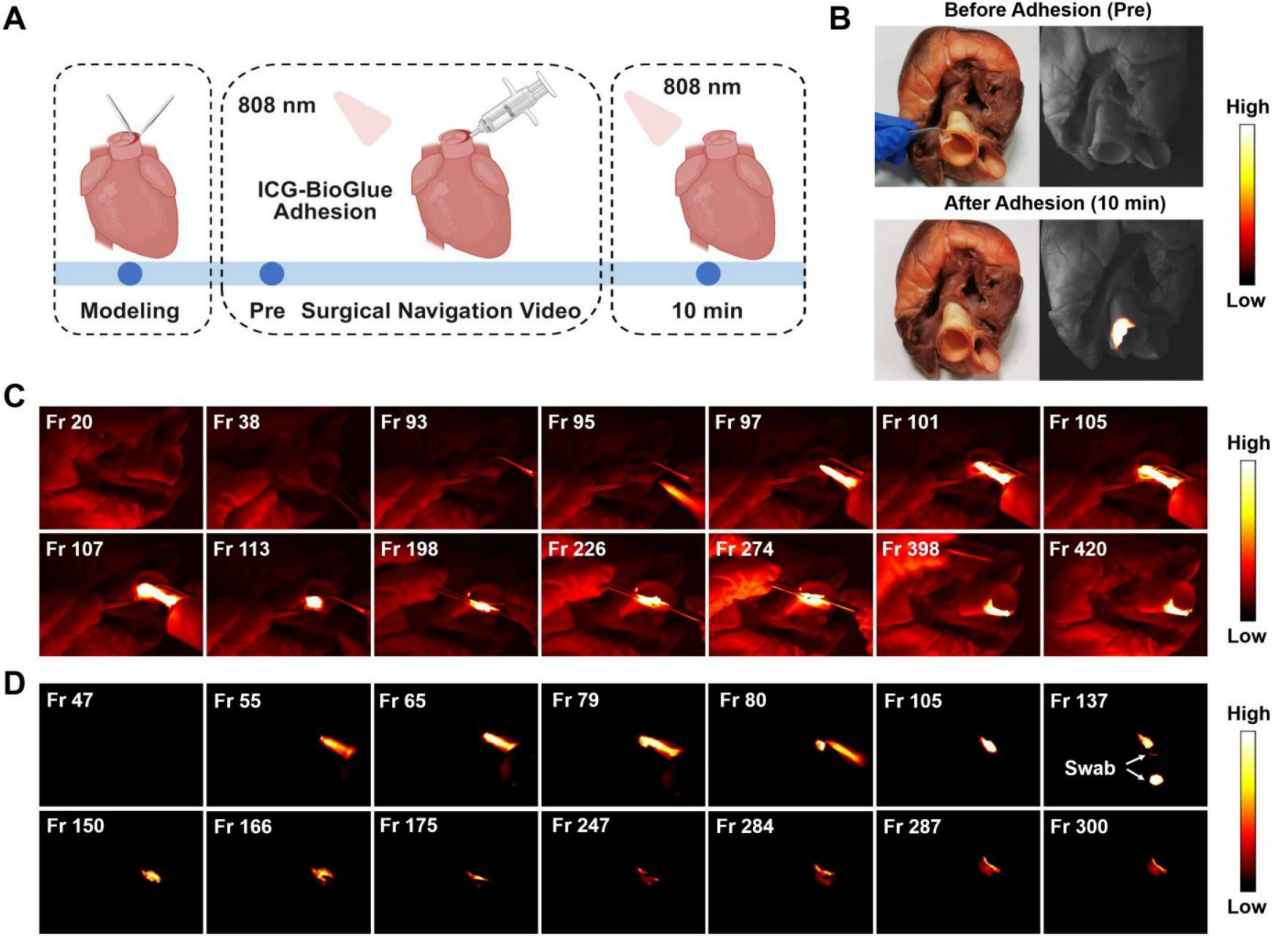
*Ex vivo* aortic dissection repair surgery guided by fluorescence imaging. (**A**) Schematic illustration of the aortic dissection repair surgery guided by fluorescence imaging using ICG-BioGlue adhesive. (**B**) Photographs and NIR-II fluorescence images of the aortic root before and after the adhesion. (**C**) The frames of the surgical navigation video with the illumination by a white light source. (**D**) The frames of the surgical navigation video without the illumination by a white light source.

The visualized wound adhesion of the ICG-BioGlue adhesive was explored using NIR-II fluorescence imaging. The *ex vivo* aortic dissection repair model was selected, which was the FDA-approved indication for BioGlue adhesive [9]. The intima and media of the aortic root in pig hearts were separated to create a cavity, and the ICG-BioGlue adhesive (100 μL) was visually injected to bond the cavity via real-time NIR-II fluorescence imaging. Finally, NIR-II fluorescence images along with surgical navigation videos were gathered for analysis (Figure 4A). The images of the aortic root, including both regular photos and NIR-II fluorescence, demonstrated the disappearance of the cavity following the injection of ICG-BioGlue adhesive. Additionally, the adhesive area was delineated by intense NIR-II fluorescence (Figure 4B). The whole surgical process guided via NIR-II fluorescence imaging with or without being illuminated by a white light source was recorded as videos. The video frames depicted the bonding process of ICG-BioGlue adhesive to the cavity, enabling observation of the range and shape of the adhesive, thus preventing leaks into adjacent areas (Figure 4C and D and Supplementary Movies S1 and S2). Meanwhile, we performed a quantitative analysis of fluorescence intensity based on the NIR-II fluorescence images acquired during the aortic root adhesion process under NIR-II fluorescence guidance. The results showed a marked increase in fluorescence intensity within the adhesion region compared with that before surgery, clearly delineating the area of adhesive application (Supplementary Figure S10). The above experiments showed the bright fluorescence and good adhesion performance of ICG-BioGlue adhesive for wound adhesion visualization guided by fluorescence imaging in aortic dissection repair surgery *ex vivo*.

### *In vivo* hepatic injury adhesion visualization guided by fluorescence imaging

**Figure 5:**
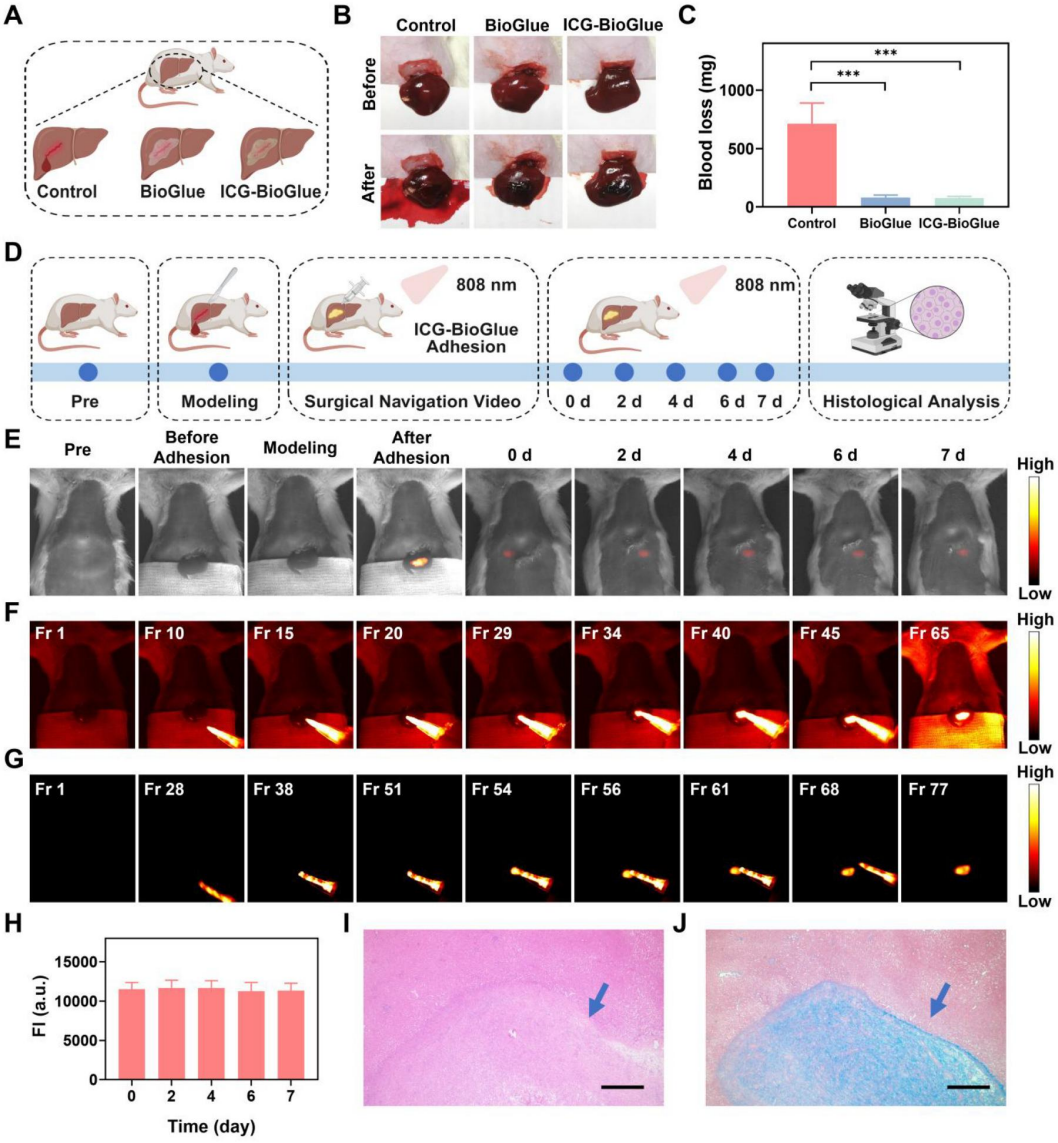
*In vivo* hepatic injury adhesion visualization guided by fluorescence imaging. (**A**) Schematic illustration of the evaluation of liver hemostasis using ICG-BioGlue and BioGlue adhesive. (**B**) Photographs and (**C**) quantitative analysis of the blood loss using ICG-BioGlue and BioGlue adhesive (*n* = 3). (**D**) Schematic illustration of visualization and monitoring of the liver wound adhesion under the guidance of fluorescence imaging using ICG-BioGlue adhesive. (**E**) NIR-II fluorescence images of rats undergoing wound adhesion surgery preoperatively, intraoperatively, and postoperatively. (**F**) The frames of the surgical navigation video with the illumination by a white light source. (**G**) The frames of the surgical navigation video without the illumination by a white light source. (**H**) NIR-II fluorescence intensity of (**E**) (*n* = 6). (**I**) H&E staining image and (**J**) Masson’s staining image of the liver wound. Blue arrow: healing wound. Scale bars, 500 µm. FI, fluorescence intensity.

Hemostasis of major organs such as the liver was also one of the clinical applications of BioGlue adhesive. The hepatic bleeding model was established to assess the performance of the ICG-BioGlue adhesive for visual hepatic injury healing using NIR-II fluorescence imaging [9]. Following the building of a liver incision measuring 10 mm in length and 3 mm in depth, each adhesive was individually administered for wound adhesion to compare the hemostatic ability of ICG-BioGlue and BioGlue adhesive (Figure 5A). A hepatic bleeding model without hemostatic treatment was set as the control. Following analysis of bleeding images and quantification, it was determined that both ICG-BioGlue and BioGlue adhesive exhibited outstanding hemostatic efficacy compared to the control group (Figure 5B and C). Subsequently, we applied the ICG-BioGlue adhesive for visualizing liver wound adhesion using NIR-II fluorescence imaging, capturing the NIR-II fluorescence images and surgical navigation videos. The NIR-II fluorescence imaging of the rat abdomen was conducted at different time points (0, 2, 4, 6 and 7 days), and the liver was collected on day 7 for hematoxylin and eosin (H&E) and Masson’s staining to evaluate the wound healing status (Figure 5D). The outline and bright NIR-II fluorescence signal of the wound adhesion were presented in the photos and NIR-II fluorescence imaging during the bonding process (Figure 5E and Supplementary Figure S11). The video and frames of the NIR-II fluorescence imaging-guided surgery (illuminated with or without a white light source) showed the operation of wound adhesion in the liver with the ICG-BioGlue adhesive. The NIR-II fluorescence signal range of the adhesive matched the wound shape without any adhesive migration (Figure 5F and G and Supplementary Movies S3 and S4). In addition, we performed a quantitative analysis of the fluorescence intensity from the NIR-II fluorescence images acquired during the NIR-II fluorescence-guided hepatic wound adhesion process. A significant increase in fluorescence intensity was observed within the adhesive region, indicating that the hepatic wound was effectively sealed (Supplementary Figure S12). The NIR-II fluorescence images were captured within 7 days post-operation, and the NIR-II fluorescence intensity was stably located in the liver, reflecting the excellent implantation performance and fluorescence stability of the ICG-BioGlue adhesive *in vivo* (Figure 5E and H). The H&E staining showed the accumulation of inflammatory cells at the incision, and Masson’s staining exhibited collagen deposition in the wound, indicating gradual healing (Figure 5I and J). These findings illustrate the effective application of the ICG-BioGlue adhesive for achieving visualized liver hemostasis, alongside real-time monitoring of adhesive placement through NIR-II fluorescence imaging.

Since accidental leakage of BioGlue may lead to the formation of microemboli, posing serious health risks to patients, it is therefore of great importance to evaluate unintended BioGlue leakage. We further investigated whether trace leakage of ICG-BioGlue adhesive could be detected under NIR-II fluorescence imaging guidance. Cubic ICG-BioGlue adhesive blocks of different sizes (with edge lengths of 1, 2, 3, 4 and 5 mm) were prepared. During NIR-II fluorescence-guided hepatic hemostasis experiments, these cubes were placed on the liver surface to simulate emboli formed by adhesive leakage. The results showed that even the 1-mm cubic block could be clearly visualized, demonstrating that trace leakage of ICG-BioGlue adhesive can be effectively monitored under NIR-II fluorescence imaging guidance (Supplementary Figure S13).

### *In vivo* kidney injury adhesion visualization guided by fluorescence imaging

**Figure 6:**
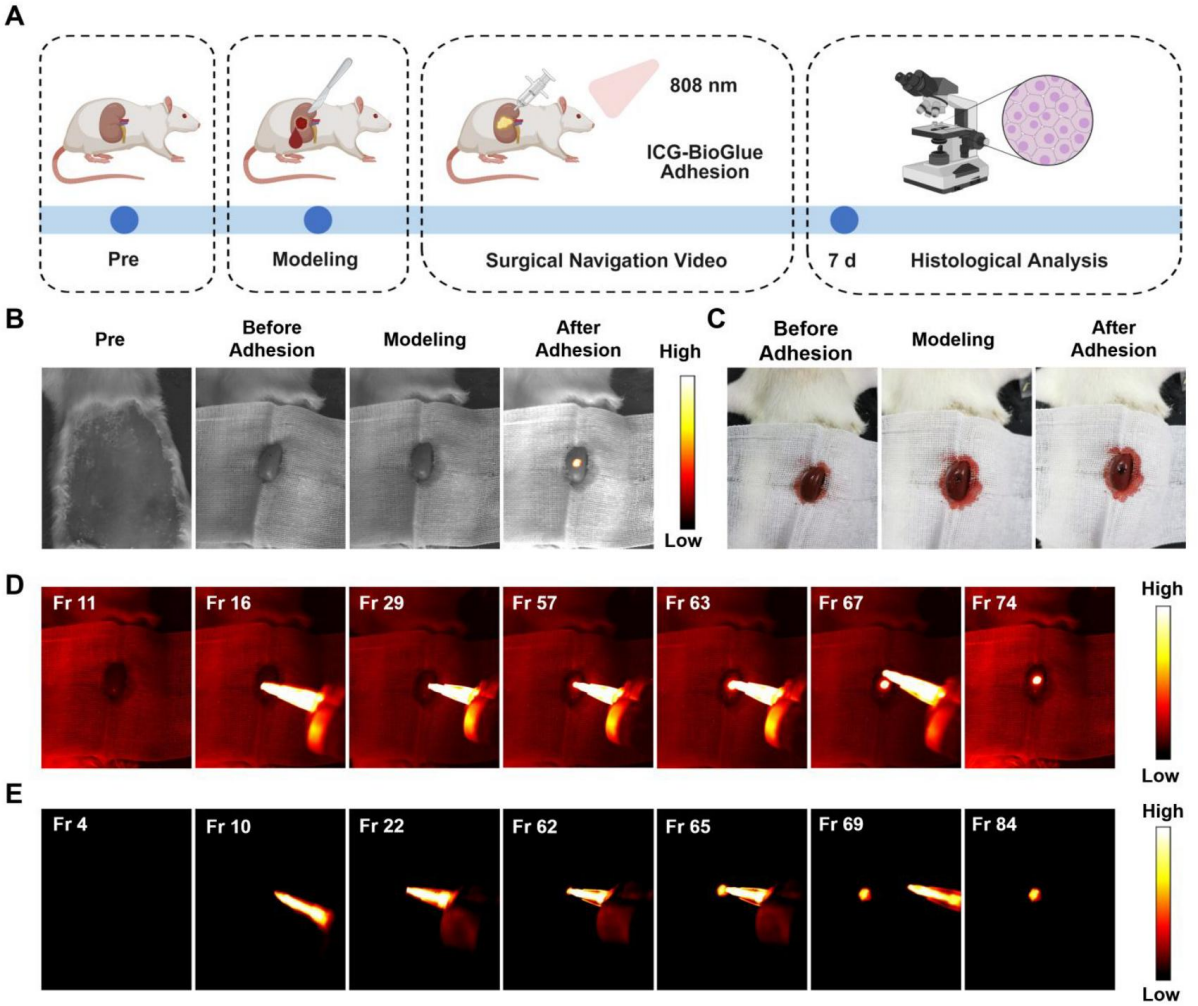
*In vivo* kidney injury adhesion visualization guided by fluorescence imaging. (**A**) Schematic illustration of visualization and monitoring of the kidney wound adhesion under the guidance of fluorescence imaging using ICG-BioGlue adhesive. (**B**) NIR-II fluorescence images and (**C**) photographs of rats undergoing wound adhesion surgery. (**D**) The frames of the surgical navigation video with the illumination by a white light source. (**E**) The frames of the surgical navigation video without the illumination by a white light source.

For further investigating the *in vivo* hemostatic performance of the ICG-BioGlue adhesive, the kidney bleeding model was chosen, as the BioGlue adhesive was also clinically suitable for hemostasis in nephron-sparing surgeries [9]. Circular wounds with a diameter of 2 mm were created on the exposed left kidneys of rats, followed by the completion of fluorescence imaging-guided wound adhesion and hemostasis procedures. Moreover, histopathological staining of the kidneys was employed to assess the wound healing conditions facilitated by the ICG-BioGlue adhesive, after 7 days post-operation (Figure 6A). The wound adhesion process was guided by NIR-II fluorescence imaging, during which the images and photos showed good wound adhesion and hemostasis of the ICG-BioGlue adhesive (Figure 6B and C). The entire adhesion process was recorded through NIR-II fluorescence imaging with or without the white light source irradiation, and the adhesive covered the incision well without leaking to normal tissues, which showed the visibility and controllability of the ICG-BioGlue adhesive injection (Figure 6D and E and Supplementary Movies S5 and S6). We also conducted a quantitative analysis of the fluorescence intensity from the NIR-II fluorescence images acquired during the renal wound sealing procedure. Compared with the pre-adhesion state, a markedly increased fluorescence intensity was observed at the wound site, indicating that the renal wound was effectively sealed (Supplementary Figure S14). The H&E staining and Masson’s staining exhibited a healing wound with inflammatory cells and collagen deposition in the wound, proving that the wound was during the healing process (Supplementary Figure S15). These results indicated that NIR-II fluorescence imaging of ICG-BioGlue adhesive could visually guide the adhesion of renal wounds, preventing potential damage caused by ICG-BioGlue adhesive leakage into normal tissues.

### *In vivo* ICG-BioGlue fragments removal surgery guided by fluorescence imaging

**Figure 7:**
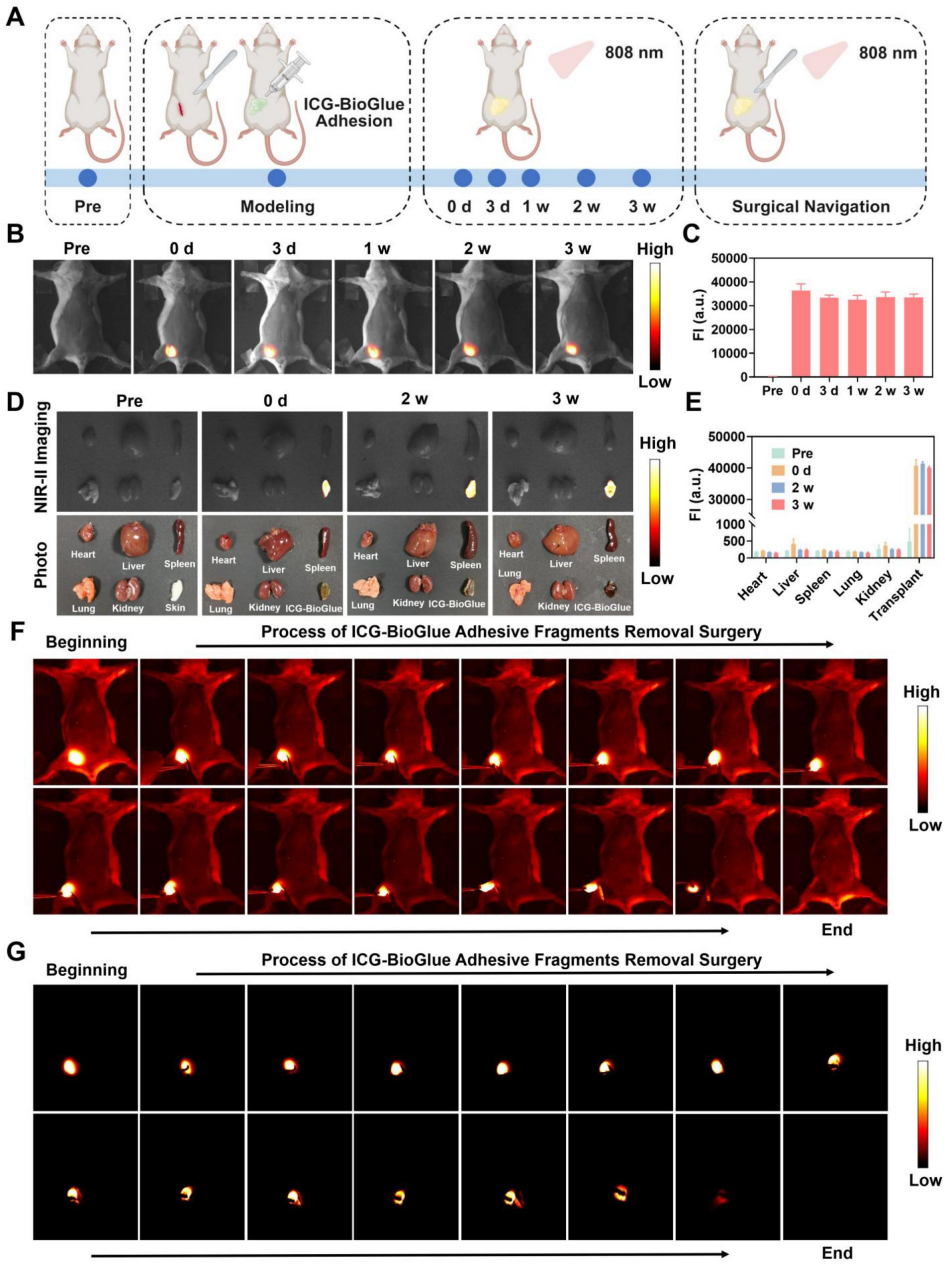
*In vivo* ICG-BioGlue adhesive fragments removal surgery guided by fluorescence imaging. (**A**) Schematic illustration of *in vivo* adhesive monitoring and surgery for adhesive fragment removal guided by fluorescence imaging using ICG-BioGlue adhesive. (**B**) NIR-II fluorescence images and (**C**) fluorescence intensity of ICG-BioGlue adhesive *in vivo* (*n* = 6). (**D**) *Ex vivo* NIR-II fluorescence images and (**E**) fluorescence intensity of the main organs after treatment with ICG-BioGlue adhesive (*n* = 3). (**F**) NIR-II fluorescence images of mice during surgery for ICG-BioGlue adhesive fragments removal with the illumination by a white light source. (**G**) NIR-II fluorescence images of mice during surgery for ICG-BioGlue adhesive fragments removal without the illumination by a white light source. FI, fluorescence intensity.

In clinical applications, BioGlue adhesive might result in local compression of normal structures or detachment, prompting the need for additional surgeries to eliminate adhesive residues. For example, in spinal dura repair surgeries, the application of BioGlue adhesive might lead to nerve compression due to inflammatory reactions, requiring immediate surgical removal of the adhesive [9]. However, traditional adhesives lack specific visualization capabilities, increasing the risk of adhesive residue. Therefore, to validate the ability of ICG-BioGlue adhesive for fluorescence imaging to guide surgical removal of adhesive fragments, we injected ICG-BioGlue adhesive into the right groin incision of mice and monitored the implantation status in real-time. Subsequently, 3 weeks later, we conducted fluorescence-guided visual surgery to remove the adhesive fragments (Figure 7A). The NIR-II fluorescence images and fluorescence intensity analysis obtained at various time points over 3 weeks revealed consistent fluorescence intensity of the ICG-BioGlue adhesive *in vivo*, thus confirming its excellent fluorescence stability (Figure 7B and C). *Ex vivo* NIR-II fluorescence imaging and fluorescence intensity analysis of the major organs and the adhesive fragments extracted from experimental mice indicated that nearly all fluorescence signals were localized at the adhesive implantation site, with no leakage of ICG-BioGlue adhesive observed in other organs (Figure 7D and E). 3 weeks after the surgery, the adhesive fragments removal surgery was performed under the guidance of fluorescence imaging (with or without white light irradiation). Due to the bright and consistent fluorescence emission of the ICG-BioGlue adhesive, the adhesive fragments located in the right inguinal area of the mouse could be entirely excised without any remaining NIR-II fluorescence signal (Figure 7F and G). Subsequently, we performed a quantitative analysis of the fluorescence intensity from the NIR-II fluorescence images acquired during the adhesive removal procedure. A significant decrease in fluorescence intensity was observed, indicating that the adhesive fragments had been completely removed (Supplementary Figure S16). These findings indicated that the ICG-BioGlue adhesive maintained stable NIR-II fluorescence *in vivo*, making it suitable for guiding the surgical removal of adhesive fragments through fluorescence imaging, thereby effectively reducing the risk of adhesive residue *in vivo*.

## Conclusion

In summary, we have proposed an NIR-II fluorescence-visible ICG-BioGlue adhesive for precision surgical adhesion with intraoperative leakage prevention. ICG-BioGlue adhesive is synthesized by straightforwardly mixing FDA-approved ICG and BioGlue adhesive based on non-covalent interaction. The ICG-BioGlue adhesive exhibits bright NIR-II fluorescence, nearly 100% fluorescence labeling efficiency, stable fluorescence over a period of up to 3 months, and excellent biocompatibility. The intense NIR-II fluorescence endows ICG-BioGlue adhesive with the capability for real-time visualization and precise control of the injection site during surgery, thereby effectively preventing leakage and potential complications. Under the guidance of high-sensitivity NIR-II fluorescence imaging, we successfully achieved precise wound adhesion in aortic dissection repairs *in vitro* and liver or kidney surgeries *in vivo*. Furthermore, fluorescence imaging guidance facilitates the efficient removal of adhesive residues in cases of localized compression symptoms. Given its extremely simple synthesis method and excellent biocompatibility, ICG-BioGlue adhesive, composed entirely of FDA-approved drugs, demonstrates significant clinical translational potential. Our proposed leakage-preventing strategy is expected to substantially advance the precise, safe, and widespread use of bioadhesives.

## Supplementary Material

rbaf131_Supplementary_Data

## Data Availability

Data will be made available upon reasonable request.

## References

[1] Bal-Ozturk A , Cecen B, Avci-Adali M, Topkaya SN, Alarcin E, Yasayan G, Ethan YC, Bulkurcuoglu B, Akpek A, Avci H, Shi K, Shin SR, Hassan S. Tissue adhesives: from research to clinical translation. Nano Today 2021;36:101049. 33425002 10.1016/j.nantod.2020.101049 PMC7793024

[2] Chen Y , Chai M, Xuan C, Lin J, Yang H, Li C, Xie M, Ostrovidov S, Shi X, Mao C. Tuning the properties of surgical polymeric materials for improved soft-tissue wound closure and healing. Prog Mater Sci 2024;143:101249.

[3] Rathi S , Saka R, Domb AJ, Khan W. Protein‐based bioadhesives and bioglues. Polymers for Advanced Techs 2019;30:217–34.

[4] Bao Z , Gao M, Sun Y, Nian R, Xian M. The recent progress of tissue adhesives in design strategies, adhesive mechanism and applications. Mater Sci Eng C Mater Biol Appl 2020;111:110796. 32279807 10.1016/j.msec.2020.110796

[5] Ma Z , Bao G, Li J. Multifaceted design and emerging applications of tissue adhesives. Adv Mater 2021;33:e2007663. 33956371 10.1002/adma.202007663

[6] Qiu L , Qi See AA, Steele TWJ, Kam King NK. Bioadhesives in neurosurgery: a review. J Neurosurg 2020;133:1928–38. 31731262 10.3171/2019.8.JNS191592

[7] Nam S , Mooney D. Polymeric tissue adhesives. Chem Rev 2021;121:11336–84. 33507740 10.1021/acs.chemrev.0c00798

[8] Dhandapani V , Ringuette V, Desrochers M, Sirois M, Vermette P. Composition, host responses and clinical applications of bioadhesives. J Biomed Mater Res B Appl Biomater 2022;110:2779–97. 35748414 10.1002/jbm.b.35113

[9] Han GY , Hwang SK, Cho KH, Kim HJ, Cho CS. Progress of tissue adhesives based on proteins and synthetic polymers. Biomater Res 2023;27:57. 37287042 10.1186/s40824-023-00397-4 PMC10249221

[10] Carrel T , Maurer M, Tkebuchava T, Niederhäuser U, Schneider J, Turina MI. Embolization of biologic glue during repair of aortic dissection. Ann Thorac Surg 1995;60:1118–20. 7574966 10.1016/0003-4975(95)97585-b

[11] LeMaire SA , Carter SA, Won T, Wang X, Conklin LD, Coselli JS. The threat of adhesive embolization: bioGlue leaks through needle holes in aortic tissue and prosthetic grafts. Ann Thorac Surg 2005;80:106–10. 15975350 10.1016/j.athoracsur.2005.02.004

[12] Suzuki S , Masuda M, Imoto K. The use of surgical glue in acute type a aortic dissection. Gen Thorac Cardiovasc Surg 2014;62:207–13. 24254987 10.1007/s11748-013-0343-0

[13] Kitamura H , Tamaki M, Kawaguchi Y. Surgical glue-induced left main trunk stenosis removed by directional coronary atherectomy. Interact Cardiovasc Thorac Surg 2022;34:162–4. 34999798 10.1093/icvts/ivab227 PMC8932500

[14] Yamazaki K , Maeda T, Sakamoto Y, Onishi Y. Pacemaker implantation improves mechanical valve mobility. J Cardiothorac Vasc Anesth 2023;37:2164–8. 10.1053/j.jvca.2023.06.034 37438179

[15] Achneck HE , Sileshi B, Jamiolkowski RM, Albala DM, Shapiro ML, Lawson JH. A comprehensive review of topical hemostatic agents: efficacy and recommendations for use. Ann Surg 2010;251:217–28. 20010084 10.1097/SLA.0b013e3181c3bcca

[16] Lauvin MA , Zemmoura I, Cazals X, Cottier JP. Delayed cauda equina compression after spinal dura repair with BioGlue: magnetic resonance imaging and computed tomography aspects of two cases of “glue-oma”. Spine J 2015;15:e5-8–e8. 10.1016/j.spinee.2014.09.012 25264182

[17] Taboada GM , Yang K, Pereira MJN, Liu SS, Hu Y, Karp JM, Artzi N, Lee Y. Overcoming the translational barriers of tissue adhesives. Nat Rev Mater 2020;5:310–29.

[18] Yokoi M , Fujita H, Ogawa T, Ito T, Seo Y, Suda H, Ohte N. Intravascular ultrasound findings of BioGlue surgical adhesive coronary embolism after ascending aorta replacement. JACC Cardiovasc Interv 2021;14:e39–e41. 33516693 10.1016/j.jcin.2020.11.018

[19] Altorfer FCS , Sutter R, Farshad M, Spirig JM, Farshad-Amacker NA. MRI appearance of adjunct surgical material used in spine surgery. Spine J 2022;22:75–83. 34284130 10.1016/j.spinee.2021.07.009

[20] Hamaguchi Y , Enomoto S, Kondo H, Tamura T, Aaysha Cader F, Bahaa H, Karanasos A, Kadavath S, Sener YZ, Abdullah A. In vivo optical coherence tomography visualisation of coronary artery embolism caused by BioGlue in a middle-aged woman with Marfan syndrome who underwent the Bentall procedure: a case report. Eur Heart J Case Rep 2023;7:ytad585. 38046217 10.1093/ehjcr/ytad585 PMC10689047

[21] Carr JA , Franke D, Caram JR, Perkinson CF, Saif M, Askoxylakis V, Datta M, Fukumura D, Jain RK, Bawendi MG, Bruns OT. Shortwave infrared fluorescence imaging with the clinically approved near-infrared dye indocyanine green. Proc Natl Acad Sci U S A 2018;115:4465–70. 29626132 10.1073/pnas.1718917115 PMC5924901

[22] Cai Y , Wei Z, Song C, Tang C, Han W, Dong X. Optical nano-agents in the second near-infrared window for biomedical applications. Chem Soc Rev 2019;48:22–37. 30444505 10.1039/c8cs00494c

[23] Mu J , Xiao M, Shi Y, Geng X, Li H, Yin Y, Chen X. The chemistry of organic contrast agents in the NIR‐II window. Angew Chem Int Ed Engl 2022;61:e202114722. 34873810 10.1002/anie.202114722

[24] Xu R , Jiao D, Long Q, Li X, Shan K, Kong X, Ou H, Ding D, Tang Q. Highly bright aggregation-induced emission nanodots for precise photoacoustic/NIR-II fluorescence imaging-guided resection of neuroendocrine neoplasms and sentinel lymph nodes. Biomaterials 2022;289:121780. 36088677 10.1016/j.biomaterials.2022.121780

[25] Yang Y , Xie Y, Zhang F. Second near-infrared window fluorescence nanoprobes for deep-tissue in vivo multiplexed bioimaging. Adv Drug Deliv Rev 2023;193:114697. 36641080 10.1016/j.addr.2023.114697

[26] Zhang X , Shen S, Liu D, Li X, Shi W, Ma H. Combination of changeable π-conjugation and hydrophilic groups for developing water-soluble small-molecule NIR-II fluorogenic probes. Chem Sci 2023;14:2928–34. 36937580 10.1039/d3sc00355h PMC10016431

[27] Wang F , Zhong Y, Bruns O, Liang Y, Dai H. In vivo NIR-II fluorescence imaging for biology and medicine. Nat Photon 2024;18:535–47.

[28] Wang Y , Zhou D, Ma H, Liu D, Liang Y, Zhu S. An ultra-small organic dye nanocluster for enhancing NIR-II imaging-guided surgery outcomes. Eur J Nucl Med Mol Imaging 2024;51:2941–52. 38581443 10.1007/s00259-024-06702-0

[29] Zhang Z , Du Y, Shi X, Wang K, Qu Q, Liang Q, Ma X, He K, Chi C, Tang J, Liu B, Ji J, Wang J, Dong J, Hu Z, Tian J. NIR-II light in clinical oncology: opportunities and challenges. Nat Rev Clin Oncol 2024;21:449–67. 38693335 10.1038/s41571-024-00892-0

[30] Liu S , Chen C, Li Y, Zhang H, Liu J, Wang R, Wong STH, Lam JWY, Ding D, Tang BZ. Constitutional isomerization enables bright NIR-II AIEgen for brain-inflammation imaging. Adv Funct Mater 2019;30:1908125.

[31] Zhu S , Tian R, Antaris AL, Chen X, Dai H. Near-infrared-II molecular dyes for cancer imaging and surgery. Adv Mater 2019;31:e1900321. 31025403 10.1002/adma.201900321 PMC6555689

[32] Hu Z , Fang C, Li B, Zhang Z, Cao C, Cai M, Su S, Sun X, Shi X, Li C, Zhou T, Zhang Y, Chi C, He P, Xia X, Chen Y, Gambhir SS, Cheng Z, Tian J. First-in-human liver-tumour surgery guided by multispectral fluorescence imaging in the visible and near-infrared-I/II windows. Nat Biomed Eng 2020;4:259–71. 31873212 10.1038/s41551-019-0494-0

[33] Wang S , Li B, Zhang F. Molecular fluorophores for deep-tissue bioimaging. ACS Cent Sci 2020;6:1302–16. 32875073 10.1021/acscentsci.0c00544 PMC7453417

[34] Xu W , Wang D, Tang BZ. NIR-II AIEgens: a Win-Win integration towards bioapplications. Angew Chem Int Ed Engl 2021;60:7476–87. 32515530 10.1002/anie.202005899

[35] He T , Jiang C, He J, Zhang Y, He G, Wu J, Lin J, Zhou X, Huang P. Manganese-dioxide-coating-instructed plasmonic modulation of gold nanorods for activatable duplex-imaging-guided NIR-II photothermal-chemodynamic therapy. Adv Mater 2021;33:e2008540. 33645863 10.1002/adma.202008540

[36] Zhang Z , He K, Chi C, Hu Z, Tian J. Intraoperative fluorescence molecular imaging accelerates the coming of precision surgery in China. Eur J Nucl Med Mol Imaging 2022;49:2531–43. 35230491 10.1007/s00259-022-05730-y PMC9206608

[37] Pan W , Rafiq M, Haider W, Guo Y, Wang H, Xu M, Yu B, Cong H, Shen Y. Recent advances in NIR-II fluorescence/photoacoustic dual-modality imaging probes. Coordin Chem Rev 2024;514:215907.

[38] Li C , Chen G, Zhang Y, Wu F, Wang Q. Advanced fluorescence imaging technology in the near-Infrared-II window for biomedical applications. J Am Chem Soc 2020;142:14789–804. 32786771 10.1021/jacs.0c07022

[39] Lei Z , Zhang F. Molecular engineering of NIR-II fluorophores for improved biomedical detection. Angew Chem Int Ed Engl 2021;60:16294–308. 32780466 10.1002/anie.202007040

[40] Park GK , Kim SH, Kim K, Das P, Kim BG, Kashiwagi S, Choi HS, Hwang NS. Dual-channel fluorescence imaging of hydrogel degradation and tissue regeneration in the brain. Theranostics 2019;9:4255–64. 31285760 10.7150/thno.35606 PMC6599647

[41] He SN , Xie F, Su WY, Luo HB, Chen DL, Cai J, Hong XC. Anti-inflammatory salidroside delivery from chitin hydrogels for NIR-II image-guided therapy of atopic dermatitis. JFB 2023;14:150. 36976074 10.3390/jfb14030150 PMC10058600

[42] Sun LH , Ouyang J, She ZP, Li R, Zeng F, Yao ZC, Wu SZ. Injectable-hydrogel-based tissue sealant for hemostasis, bacteria inhibition, and pro-angiogenesis in organ bleeding wounds and therapeutic outcome monitoring via NIR-II optical imaging. Adv Healthcare Mater 2024;13:16. 10.1002/adhm.202303997 38281086

[43] Wang M , Lin B, Chen YT, Liu HY, Ju ZY, Lv RC. Fluorescence-recovered wearable hydrogel patch for in vitro detection of glucose based on rare-earth nanoparticles. ACS Biomater Sci Eng 2024;10:1128–38. 38221709 10.1021/acsbiomaterials.3c01682

[44] Yang Y , He SN, Wang WM, Lu YW, Ren BT, Dan C, Ji Y, Yu R, Ju XP, Qiao X. NIR-II image-guided wound healing in hypoxic diabetic foot ulcers: the potential of ergothioneine-luteolin-chitin hydrogels. Macromol Rapid Commun 2024;45:12. 10.1002/marc.202400528 39422630

[45] Yu XH , Zeng LA, Yang XY, Ren ZL, Dong XM, Meng G, Shan GG, Yan DY, Wang D, Sun F. An NIR-II absorbing injectable hydrogel for boosted photo-immunotherapy toward human papillomavirus associated cancer. Aggregate 2025;6:10.

[46] Wang HL , Li XX, Tse BWC, Yang HT, Thorling CA, Liu YX, Touraud M, Chouane JB, Liu X, Roberts MS, Liang XW. Indocyanine green-incorporating nanoparticles for cancer theranostics. Theranostics 2018;8:1227–42. 29507616 10.7150/thno.22872 PMC5835932

[47] Chen H , Cheng HW, Dai QX, Cheng Y, Zhang Y, Li DF, Sun Y, Mao JS, Ren K, Chu CC, Liu G. A superstable homogeneous lipiodol-ICG formulation for locoregional hepatocellular carcinoma treatment. J Control Release 2020;323:635–43. 32302761 10.1016/j.jconrel.2020.04.021

[48] Xiong YF , He P, Zhang Y, Chen H, Peng YS, He P, Tian J, Cheng HW, Liu G, Li JD. Superstable homogeneous lipiodol-ICG formulation: initial feasibility and first-in-human clinical application for ruptured hepatocellular carcinoma. Regen Biomater 2023;10:rbac106. 36683740 10.1093/rb/rbac106 PMC9847516

[49] Grigoryan K , Shilajyan H, Savvaidis I, Mkhitaryan L, Zatikyan A. Effect of bovine serum albumin on gallic acid and tannic acid radical scavenging properties and binding kinetics with 2,2-diphenyl-1-picrylhydrazyl. Process Biochem 2024;141:30–8.

[50] Lović J , Stevanović S, Andjelković B, Petrović S, Vuković D, Prlainović N, Mijin D, Nikolić ND, Ivić MA. Electrochemical glucose biosensor with the characterization of surface morphology and content of glucose oxidase-glutaraldehyde-cysteine layers on gold electrode. Int J Electrochem Sci 2018;13:12340–8.

[51] Antaris AL , Chen H, Diao S, Ma Z, Zhang Z, Zhu S, Wang J, Lozano AX, Fan Q, Chew L, Zhu M, Cheng K, Hong X, Dai H, Cheng Z. A high quantum yield molecule-protein complex fluorophore for near-infrared II imaging. Nat Commun 2017;8:15269. 28524850 10.1038/ncomms15269 PMC5454457

[52] Li D , Qu C, Liu Q, Wu Y, Hu X, Qian K, Chang B, He S, Yuan Y, Li Y, Ko T, Yu A, Cheng Z. Monitoring the real-time circulatory system-related physiological and pathological processes in vivo using a multifunctional NIR-II probe. Adv Funct Mater 2019;30:1906343.

[53] Tian R , Zeng Q, Zhu SJ, Lau J, Chandra S, Ertsey R, Hettie KS, Teraphongphom T, Hu ZB, Niu G, Kiesewetter DO, Sun HT, Zhang XD, Antaris AL, Brooks BR, Chen XY. Albumin-chaperoned cyanine dye yields superbright NIR-II fluorophore with enhanced pharmacokinetics. Sci Adv 2019;5:eaaw0672. 31548981 10.1126/sciadv.aaw0672 PMC6744268

[54] Sakka SG. Assessing liver function. Curr Opin Crit Care 2007;13:207–14. 17327744 10.1097/MCC.0b013e328012b268

[55] Moody ED , Viskari PJ, Colyer CL. Non-covalent labeling of human serum albumin with indocyanine green: a study by capillary electrophoresis with diode laser-induced fluorescence detection. J Chromatogr B Biomed Sci Appl 1999;729:55–64. 10410927 10.1016/s0378-4347(99)00121-8

[56] Li XD , Fu Y, Ma LN, Wang ZX, Zhang HM. Spectrometric study on the interaction of indocyanine green with human serum albumin. Chem Res Chin Univ 2016;32:343–7.

