# Supplementary material for: Fabrication of an NIR-II fluorescence-visible indocyanine green-BioGlue for precision surgical adhesion with intraoperative leakage prevention
Source: Regen Biomater. 2025 Dec 24;13:rbaf131. doi: 10.1093/rb/rbaf131 (PMC12869796; doi:10.1093/rb/rbaf131)
Supplement: rbaf131_Supplementary_Data [file rbaf131_supplementary_data.zip › 28-Dec-2025_081325_Supplementary_Material.zip!/Revised Supplementary Material.docx]

**Supplementary Material**

**Fabrication of an NIR-II Fluorescence-Visible Indocyanine Green-BioGlue for Precision Surgical Adhesion with Intraoperative Leakage Prevention**

Xinyu Feng ^1,2,#^, Ruihan Liu ^1,#^, Cai Zhang ^3,*^, Ran Cheng ^1^, Dianxun Fu ^4^, Shao-Kai Sun ^1,*^, Junping Wang ^4,*^, and Guohe Wang ^1,*^

^1^ School of Medical Imaging, Division of Medical Technology, Tianjin Key Laboratory of Functional Imaging, Tianjin Medical University, Tianjin 300203, China Email: shaokaisun@tmu.edu.cn, wangguohe@tmu.edu.cn

^2^ Department of Breast Imaging, Tianjin Medical University Cancer Institute & Hospital, National Clinical Research Center for Cancer, Tianjin's Clinical Research Center for Cancer, Key Laboratory of Breast Cancer Prevention and Therapy, Tianjin Medical University, Ministry of Education, Key Laboratory of Cancer Prevention and Therapy, Tianjin 300060, China

^3^ Department of Radiology, Tianjin Medical University Cancer Institute and Hospital, National Clinical Research Center for Cancer, Tianjin’s Clinical Research Center for Cancer, Key Laboratory of Cancer Immunology and Biotherapy, Tianjin 300060, China Email: caizhang@tmu.edu.cn

^4^ Department of Radiology, Tianjin Key Laboratory of Functional Imaging, Tianjin Medical University General Hospital, Tianjin, 300052, China Email: wangjunping_tj@163.com

*Corresponding author

^#^These authors contributed equally to this paper.

Figure S1. (A) Absorbance spectrum of ICG (5 μg/mL). (B) NIR-II fluorescence spectrum of ICG (10 μg/mL). FI: fluorescence intensity.

**Figure S2.** (A) Photographs of BSA solution and ICG-BioGlue adhesive both containing varying concentrations of ICG in upright states. (B) Photographs of BSA solution and ICG-BioGlue adhesive both containing varying concentrations of ICG in inclined states. (C) NIR-II fluorescence images of ICG-BioGlue adhesive containing varying concentrations of ICG. (D) Fluorescence intensity of images in (C) (n = 3). FI: fluorescence intensity.

**Figure S3.** Photographs of (A) BioGlue adhesive and (B) ICG-BioGlue adhesive in tensile test. (C) The maximum tension analysis of ICG-BioGlue and BioGlue adhesive (n = 3).

**Figure S4.** Rheological properties of BioGlue and ICG-BioGlue adhesive. G': storage modulus, G'': loss modulus.

**Figure S5.** (A) Photographs of ICG-BioGlue adhesive adhered to porcine skin under squeezing, stretching, and twisting. (B) Photographs showing the adhesion of ICG-BioGlue adhesive to rat organs.

**Figure S6.** Adhesion strength of ICG-BioGlue and BioGlue adhesive (n = 3).

**Figure S7.** 100 cycles of loading tests performed on the skin samples bonded with (A) BioGlue and (B) ICG-BioGlue adhesive.

**Figure S8.** Photographs of ICG-BioGlue adhesive blocks immersed in water or PBS at 4°C or 25°C for 100 days.

**Figure S9**. Cell viabilities of HUVECs treated with different concentrations of ICG-BioGlue adhesive extracts (n = 6).

**Figure S10.** A quantitative analysis of the fluorescence intensity from the NIR-II fluorescence images acquired during the NIR-II fluorescence-guided aortic root adhesion process (A) with the illumination by a white light source and (B) without the illumination by a white light source (n = 3). FI: fluorescence intensity.

**Figure S11.** Photographs of rats undergoing liver wound adhesion.

**Figure S12.** A quantitative analysis of the fluorescence intensity from the NIR-II fluorescence images acquired during the NIR-II fluorescence-guided hepatic wound adhesion process (A) with the illumination by a white light source and (B) without the illumination by a white light source (n = 3). FI: fluorescence intensity.

**Figure S13.** (A) Photographs and NIR-II images of cubic ICG-BioGlue adhesive blocks of different sizes. (B) NIR-II images of cubic ICG-BioGlue adhesive blocks of different sizes during hepatic adhesion surgery.

**Figure S14.** A quantitative analysis of the fluorescence intensity from the NIR-II fluorescence images acquired during the NIR-II fluorescence-guided renal wound adhesion process (A) with the illumination by a white light source and (B) without the illumination by a white light source (n = 3). FI: fluorescence intensity.

**Figure S15.** (A) H&E staining image and (B) Masson’s staining image of the kidney wound. Blue arrow: healing wound. Scale bars, 500 µm.

**Figure S16.** A quantitative analysis of the fluorescence intensity from the NIR-II fluorescence images acquired during the NIR-II fluorescence-guided the adhesive removal procedure (A) with the illumination by a white light source and (B) without the illumination by a white light source (n = 3). FI: fluorescence intensity.

Movie S1 (separate file). The video of the aortic dissection repair surgery guided by fluorescence imaging using ICG-BioGlue adhesive with the illumination by a white light source.

Movie S2 (separate file). The video of the aortic dissection repair surgery guided by fluorescence imaging using ICG-BioGlue adhesive without the illumination by a white light source.

Movie S3 (separate file). The video of the hepatic injury adhesion visualization guided by fluorescence imaging using ICG-BioGlue adhesive with the illumination by a white light source.

Movie S4 (separate file). The video of the hepatic injury adhesion visualization guided by fluorescence imaging using ICG-BioGlue adhesive without the illumination by a white light source.

Movie S5 (separate file). The video of the kidney injury adhesion visualization guided by fluorescence imaging using ICG-BioGlue adhesive with the illumination by a white light source.

Movie S6 (separate file). The video of the kidney injury adhesion visualization guided by fluorescence imaging using ICG-BioGlue adhesive without the illumination by a white light source.
